# Practical Workflow for Building Local Mass Spectral Libraries for Untargeted Metabolomics

**DOI:** 10.3390/metabo16060412

**Published:** 2026-06-12

**Authors:** Torbjørn Norberg Myhre, Terkel Hansen, Tetiana Lutchyn, Marie Mardal, Terje Vasskog

**Affiliations:** 1 Department of Pharmacy, UiT—The Arctic University of Norway, 9037 Tromsoe, Norway; terkel.hansen@sintef.no (T.H.); marie.mardal@sund.ku.dk (M.M.); terje.vasskog@uit.no (T.V.); 2 Department of Biotechnology and Nanomedicine, SINTEF Industry, 7034 Trondheim, Norway; 3 Department of Physics and Technology, UiT—The Arctic University of Norway, 9037 Tromsoe, Norway; ltt192725@gmail.com; 4 Department of Forensic Medicine, University of Copenhagen, 2100 Copenhagen Ø, Denmark

**Keywords:** mass spectral library, fragmentation spectra, LC-MS/MS, mzVault, compound discoverer, MSMLS

## Abstract

**Background**: Metabolite identification and annotation remain major bottlenecks in untargeted metabolomics because mass spectral features often lack sufficient specificity. High-confidence annotation requires experimental validation using authentic standards analyzed under matched chromatographic and ionization conditions, providing greater reliability than in silico predictions or database matching alone. This study aimed to develop a practical and scalable workflow for constructing a high-quality mass spectral library using a commercially available analytical standards kit. **Methods**: A total of 603 metabolites from the MSMLS kit were organized into 42 mixtures, each containing approximately 15 compounds. Mixture design was based on molecular mass and distribution coefficient values, specifically logD at pH 3.1, with a minimum logD spacing of 0.15 to improve chromatographic separation and reduce co-elution. This strategy was used to minimize the total number of injections while maintaining spectral quality. The resulting spectra were evaluated against online spectral resources and in silico fragmentation predictions. A preliminary proof-of-concept analysis was also performed using human serum samples. **Results**: Using this workflow, 471 metabolites, corresponding to approximately 78% of the standards, were successfully detected and incorporated into the spectral library. Comparison with online resources and in silico fragmentation predictions demonstrated improved spectral quality and reliability. The proof-of-concept serum analysis enabled identification of endogenous metabolites using the constructed library. In addition, the robustness and applicability of the workflow were further supported by a method validation study using metabolites derived from this library. **Conclusions**: This workflow provides a scalable strategy for constructing mass spectral libraries that balances spectral quality with analytical throughput. By using rational mixture design and authentic standards analyzed under matched experimental conditions, the approach enables substantial metabolite coverage while maintaining data reliability and minimizing experimental effort.

## 1. Introduction

Untargeted metabolomics involves the comprehensive study of metabolites within an organism to gain insight into its biological state. Metabolomics is a complex omics science because it captures dynamic chemical interactions within an organism, making it the omics layer closest to the actual phenotype. The conversion of liquid chromatography–high-resolution mass spectrometry (LC-HRMS) data into biological knowledge relies on the accurate annotation and identification of detected metabolites. However, accurate annotation and identification remain a significant bottleneck in LC-HRMS-driven untargeted metabolomics [1,2,3].

Metabolomics focuses on the complete set of small molecules (usually <1500 Da), known as the metabolome, present in cells, biofluids, tissues, or organisms [4]. The metabolome is influenced by both genetic and environmental factors, serving as a bridge between genotype and phenotype. It has the potential to uncover novel biomarkers for disease and can be utilized in personalized medicine to monitor medication responses at the metabolic level [5]. Metabolites are both substrates and products of cellular functions and can be either endogenous (produced by the host) or exogenous (originating from dietary sources, medicinal use, microorganisms such as the gut microbiota, or environmental sources) [6]. This results in variable levels of specific metabolites among individuals, depending on diet, health status, microbiota, and environmental factors. By implementing a robust study design to address these challenges, it is possible to identify patterns in the metabolome that correspond to biological or environmental factors, such as pathogenesis, which may have diagnostic or prognostic value.

As structural isomers cannot be separated by the mass spectrometer alone, and direct infusion is subject to ion suppression effects, chromatography provides an orthogonal temporal separation of features. This helps distinguish metabolites with similar *m*/*z* values and alleviates some suppression effects, resulting in more detected features and improved reproducibility. The most commonly used chromatographic methods in untargeted metabolomics are reverse-phase liquid chromatography (RPLC) and hydrophilic interaction liquid chromatography (HILIC). Their combination provides an additional orthogonal mechanism, as they differ in selectivity: RPLC retains more hydrophobic metabolites, while HILIC retains polar metabolites [7].

In LC-HRMS, a metabolite is detected as a feature defined by its mass-to-charge ratio (*m*/*z*) and corresponding retention time (RT). A single LC-HRMS untargeted metabolomics experiment may detect over 20,000 such features. However, the reported number of metabolites is usually an order of magnitude lower, illustrating the complexity of the analyzed matrices [8]. Detected features include metabolites and their isomers, typically present as multiple adducts, along with features arising from artifacts and contamination. After filtering out contamination, many features remain as structural isomers with similar RT, making accurate annotation and identification challenging. Artifacts, such as in-source fragments, further complicate the issue, as these fragments can be indistinguishable from features of metabolites of interest or may appear as unresolvable features [8,9].

Guidelines have been published on identification confidence in untargeted metabolomics, defining four identification levels [10,11]. According to these criteria, the highest confidence is achieved by analyzing authentic standards under identical conditions, as chromatographic behavior is method- and instrument-specific. Without authentic standards, it is possible to reach level 2 confidence by comparing physicochemical properties and fragmentation spectra to those in external spectral libraries. Level 3 corresponds to putative characterization based on physicochemical properties or spectral matching, while level 4 represents unknown compounds. All features, regardless of identification status, can be integrated and used for between-group comparisons; however, for findings to have clinical relevance, metabolites must be confidently identified. The inclusion of authentic standards also improves the identification confidence of compounds at levels 2–4 that are not among the standards themselves by enabling RT prediction using machine learning [12].

Online mass spectral repositories play an essential role in compound identification, offering valuable public resources such as MassBank of North America (MoNA) [13], MzCloud, LIPID Metabolites and Pathway Strategy (LIPID MAPS) [14], and the METLIN Metabolite and Chemical Entity Database (METLIN) [15]. Additionally, the European Molecular Biology Laboratory (EMBL) [16] database includes not only spectral data of reference standards but also chromatographic data and adduct information, which can aid identification when spectral data alone is insufficient. When a compound has been fragmented, spectral matching against these databases enables rapid level 2 identification, depending on the uniqueness of the fragmentation spectrum. However, there is a risk of false positives, particularly in cases involving positional isomers [17].

This work presents a practical workflow for constructing a mass spectral library of commercially available authentic standards, such as the Mass Spectrometry Metabolite Library of Standards (MSMLS). The pure (>95%) authentic standards are analyzed using four orthogonal methods consisting of RPLC and HILIC in both positive and negative ionization modes. Curation is supported by spectral similarity scoring against online MS/MS repositories and by examining the relationship between method RT and the calculated distribution coefficient at pH 3.1 (logD_3.1_).

## 2. Materials and Methods

### 2.1. Standards and Reagents

The mass spectrometry metabolite library of standards (MSMLS 230-1) was purchased from IROA technologies (Sea Girt, NJ, USA). Ammonium formate, ammonium acetate and sodium hydroxide along with LC-MS grade- methanol, acetonitrile, water, and isopropanol were purchased from Fisher Scientific (Pittsburg, PA, USA). Mixed and anonymized human serum samples from previous work were used for proof-of-concept injections.

### 2.2. Preparation of Reference Standards

The MSMLS reference standard kit comprised 603 unique metabolites (5 µg each) distributed across seven 96-well plates.

To enable efficient library construction while minimizing co-elution, 42 mixtures each containing 15 metabolites were designed, representing a compromise between analytical throughput and chromatographic resolution. Metabolites within each mixture were selected based on molecular mass and distribution coefficient (logD) to reduce the likelihood of chromatographic overlap. A minimum logD spacing of 0.15 at pH 3.1 (logD_3.1_), calculated using VCClab [18], was applied to promote RT differences under reverse-phase conditions. This threshold represents a pragmatic compromise, ensuring sufficient chromatographic separation while limiting the number of required injections.

Reference standards were dissolved and mixed using an automated liquid handling system (TECAN Fluent, Basel, Switzerland). To ensure accurate handling of small volumes of organic solvents, liquid class settings were optimized and validated prior to use. Polar and medium-polar metabolites were dissolved in 20 µL methanol:water (50:50, *v*/*v*), shaken for 1 min at 1300 rpm on a BioShake (QInstruments, Jena, Germany), followed by the addition of 80 µL water and a second shaking step under the same conditions. Apolar metabolites were dissolved in 100 µL methanol and shaken for 1 min at 1300 rpm.

Subsequently, 75 µL of each supernatant were combined into cryotubes to generate the final mixtures, which were stored until analysis. The resulting mixtures contained each metabolite at a final concentration of 3.33 µg/mL, with water content varying between 0% and 90% depending on composition.

Aliquots of each mixture were transferred into separate 96-well plates for hydrophilic interaction liquid chromatography (HILIC) and reverse-phase liquid chromatography (RPLC) analyses. Prior to injection, mixtures with high organic content were diluted with 50% LC-MS-grade water for RPLC, while all mixtures were diluted with 50% acetonitrile for HILIC to optimize chromatographic performance.

### 2.3. Preparation of Serum Samples

Serum samples were prepared by adding 450 µL of quenching solution (ACN:MeOH, 3:1, *v*/*v*) to 50 µL of human serum in Eppendorf tubes. Samples were vortexed for 20 s and centrifuged for 10 min at 10,000 RCF (4 °C). For HILIC analysis, 50 µL of the supernatant were transferred directly to injection vials. For RPLC analysis, 50 µL were transferred to vials, evaporated to dryness under nitrogen, and reconstituted in 50 µL of 10% methanol.

### 2.4. LC-MS System Suitability and Injection Strategy

Mass accuracy (*m*/*z*) was verified weekly according to the vendor’s calibration protocol, with full instrument calibration performed when verification criteria were not met.

Metabolite mixtures were injected following successful system suitability testing. A system suitability mixture (0.1 mg/L amitriptyline, arginine, doxepin, histidine, labetalol, proline, tryptophan, and quercetin) was analyzed every sixth injection to monitor instrument performance throughout the analytical sequence. MSMLS mixtures were injected at two volumes (3 µL and 10 µL) to improve metabolite coverage across a wide range of ionization efficiencies.

### 2.5. UHPLC-MS/MS Data Acquisition

UHPLC-MS/MS was performed with a Thermo Scientific Vanquish Horizon UHPLC system coupled with a Thermo Scientific Orbitrap ID-X Tribrid Mass Spectrometer (Waltham, MA, USA). Data were acquired in data-dependent acquisition (DDA) mode, with MS/MS settings optimized for spectral library generation. Autosampler temperature was maintained at 5 °C.

HESI source parameters were set as follows: sheath gas flow rate 50 (arb.), auxiliary gas 10 (arb.), sweep gas 1 (arb.), spray voltage 3.5 kV (positive mode) or 2.5 kV (negative mode), ion transfer tube temperature 325 °C, and vaporizer temperature 350 °C.

Full MS scans were acquired at a resolution of 60,000 over an *m*/*z* range of 70–800 with normalized AGC of 25%. MS/MS spectra were acquired in both Orbitrap (resolution 50,000) and ion trap modes using an isolation window of *m*/*z* 1.2 and stepped collision energies of 20, 35, and 50 eV. Dynamic exclusion settings were applied (Orbitrap: 2 scans, 5 s; ion trap: 3 scans, 3 s). The cycle time was 1.5 s.

### 2.6. Chromatographic Conditions

Chromatographic separations were performed using Waters Acquity columns (Milford, MA, USA). HILIC separations employed an Acquity BEH Amide column (100 × 2.1 mm, 1.7 µm), while RPLC separations used an Acquity HSS T3 column (150 × 2.1 mm, 1.8 µm). LC conditions are summarized in Table 1.

The HILIC ESI− gradient started at 100% B and was held for 2.5 min, followed by a linear decrease to 60% B over 6.5 min. The gradient was then held for 0.2 min before returning to initial conditions and re-equilibrated for 6 min.

The HILIC ESI+ gradient started at 100% B and was held for 2.5 min, followed by a linear decrease to 70% B over 8.5 min, before returning to initial conditions and re-equilibrated for 6 min.

The RPLC ESI− gradient started at 10% B and was held for 0.5 min, followed by a linear increase to 98% B over 13 min. The gradient was held at 98% B for 2 min, then returned to initial conditions over 0.5 min and re-equilibrated for 0.6 min.

The RPLC ESI+ gradient started at 10% B and was held for 0.5 min, followed by a linear increase to 98% B over 16 min. The gradient was held at 98% B for 2 min, then returned to initial conditions over 0.5 min and re-equilibrated for 0.6 min.

### 2.7. Proof-of-Concept Analysis and Data Processing

Proof-of-concept serum samples were analyzed using AcquireX (Thermo Scientific, Thermo Fisher Scientific, Waltham, MA, USA) deep-scan mode, consisting of five MS/MS injections and two full-scan acquisitions. Data was processed using Compound Discoverer 3.3, where detected features were matched against the constructed spectral library. All positive identifications were manually curated based on RT and fragmentation patterns. The applicability of the constructed spectral library has also been demonstrated in an independent validation study using a subset of these metabolites [19].

### 2.8. Data Curation and Spectral Evaluation

Fragmentation spectra were curated using a combination of automated detection in mzVault (Thermo Scientific, Thermo Fisher Scientific, Waltham, MA, USA) and manual inspection of raw data. For each metabolite, the highest-intensity MS/MS spectrum was generally selected to maximize signal quality. However, in cases where interference from the isolation window (1.2 Da) affected spectral clarity, lower-intensity spectra with cleaner and more representative fragmentation patterns were preferred. Spectra obtained from 10 µL injections were used when 3 µL injections did not meet quality thresholds, while retaining RT information from the 3 µL injections when available. All spectra were recalibrated to correct for potential mass measurement deviations.

Spectral quality was assessed through comparison with public repositories, including MoNA, MassBank EU, MzCloud, and EMBL. For metabolites lacking reference spectra, insilico fragmentation was performed using MetFrag (version 2.6.8) [20] to support fragment annotation.

**Figure A1:**
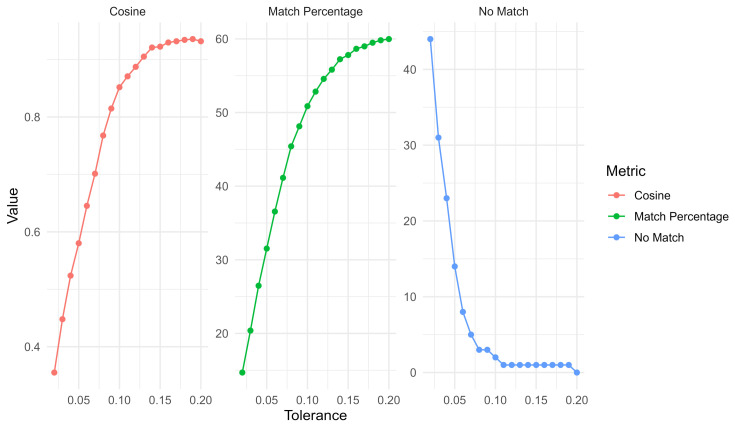
Development of matchms comparisons across different mass tolerances for entries from the RPLC library. Tolerances ranging from 0.02 to 0.20 Da (in 0.01 Da increments) were evaluated to assess the comparison of high-resolution data with low-resolution ion-trap data acquired in parallel. Cosine scores (red, **left**) show the increase in spectral similarity with increasing tolerance. Match percentage (green, **middle**) represents the proportion of fragment peaks in the high-resolution spectra that find a corresponding peak in the low-resolution data as tolerance increases. “No Match” (blue, **right**) indicates the number of library entries without a match in the low-resolution data, decreasing with increasing tolerance.

Spectral similarity comparisons were conducted using the matchms Python (version 3.13.17) package [21], employing cosine-based similarity scoring [22,23]. A mass tolerance of 0.2 Da was applied to enable comparison between high-resolution Orbitrap spectra and lower-resolution ion trap data. While this tolerance reduces mass accuracy, it allows meaningful comparison of fragmentation patterns when high-resolution reference spectra are unavailable. The effect of mass tolerance on spectral similarity between Orbitrap and ion trap data was evaluated across a range of 0.02–0.2 Da (Figure A1), demonstrating that a tolerance of 0.2 Da is required to enable meaningful comparison between high- and low-resolution spectra.

Based on empirical observations, a cosine similarity threshold of 0.85 with a minimum of four matched fragment peaks was considered sufficient for reliable spectral matching and could be used as a pragmatic criterion for automated annotation. However, to ensure the highest level of confidence and to assess the robustness of this threshold, all library entries were manually reviewed. This manual curation did not reveal clear false positives among entries meeting the threshold criteria, supporting its suitability for high-confidence spectral matching within the constructed library.

Manual curation was performed after automated spectral matching. Candidate spectra were retained when the precursor *m*/*z* matched the expected ion species, diagnostic fragment ions were present, and the spectrum exhibited a coherent fragmentation pattern consistent with reference spectra or in silico predictions. Spectra were excluded when base peak intensity was insufficient for reliable interpretation, when diagnostic ions could not be clearly distinguished from background noise, or when the spectrum was dominated by non-diagnostic peaks. Low-intensity background signals were accepted (≤20% of base peak) provided they did not interfere with the assignment of characteristic fragment ions.

**Figure 1:**
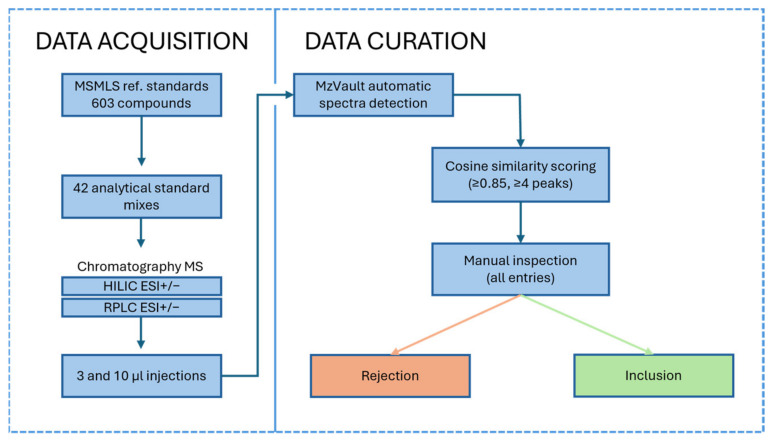
Workflow schematic for spectral library construction and curation. A total of 603 compounds were grouped into 42 analytical mixtures based on molecular mass and logD to minimize overlap. Each mixture was analyzed using four chromatographic-MS methods and two injection volumes, resulting in eight injections per mixture. Fragmentation spectra were automatically detected and evaluated using cosine similarity scoring (≥0.85, ≥4 matched peaks), followed by manual inspection of all entries prior to final inclusion or rejection in the spectral library. Green and red boxes indicate accepted and rejected compounds, respectively.

Precursor ion purity within the isolation window was evaluated, and entries were flagged when contaminant signal exceeded 10%. Chromatographic peak shape was also inspected to ensure a well-defined peak, and the relative position of MS/MS acquisition within the peak (as a percentage of peak maximum) was assessed to determine whether higher-quality spectra could be obtained, particularly for low-intensity features. The workflow for data acquisition and curation is presented in Figure 1.

## 3. Results

**Figure 2:**
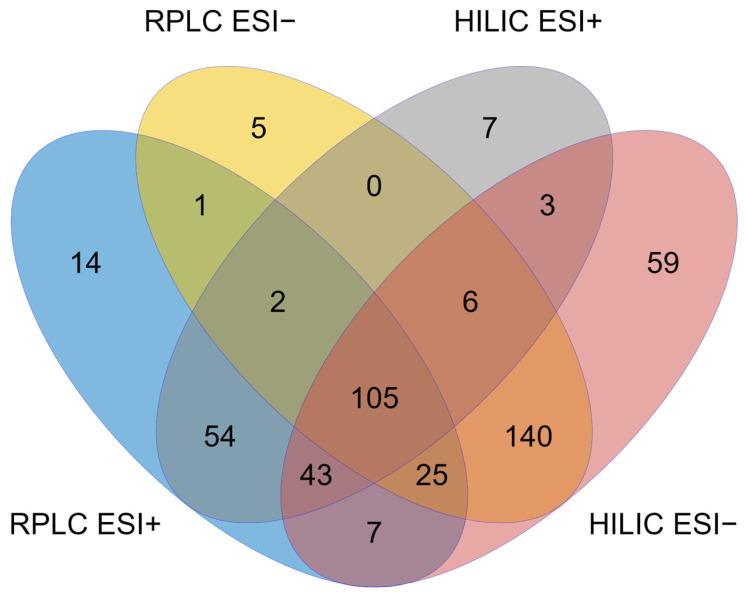
Library coverage of metabolites across chromatographic and ionization methods. The Venn diagram shows the overlap of detected metabolites between RPLC ESI+, RPLC ESI−, HILIC ESI+, and HILIC ESI− methods. A total of 105 metabolites were detected across all four methods, while substantial complementarity between methods is observed. Notably, the combination of RPLC ESI+ and HILIC ESI− covers 460 of the 471 detected metabolites (97.6%), highlighting these methods as the most complementary pair for broad metabolite coverage. Colors are used only to distinguish the four methods in the Venn diagram.

The constructed library contains 1144 MS/MS spectra with corresponding RT for 471 unique reference standards. Figure 2 shows the coverage by method and the degree of complementarity between methods. RPLC ESI+ yielded 252 compound entries, RPLC ESI− 284, HILIC ESI+ 220, and HILIC ESI− 388.

A total of 105 compounds were detected across all four analytical methods. The combination of RPLC ESI+ and HILIC ESI− covered 460 of the 471 detected compounds (97.6%).

Of the 1144 MS/MS spectra, 136 were obtained from 10 µL injections while retaining RT information from the 3 µL injections. An additional 52 entries had both RT and MS/MS spectra derived from 10 µL injections and were therefore annotated with RT uncertainty in the library.

In 84 cases, an interference peak exceeding 10% of the base peak intensity was observed within the isolation window.

**Figure A2:**
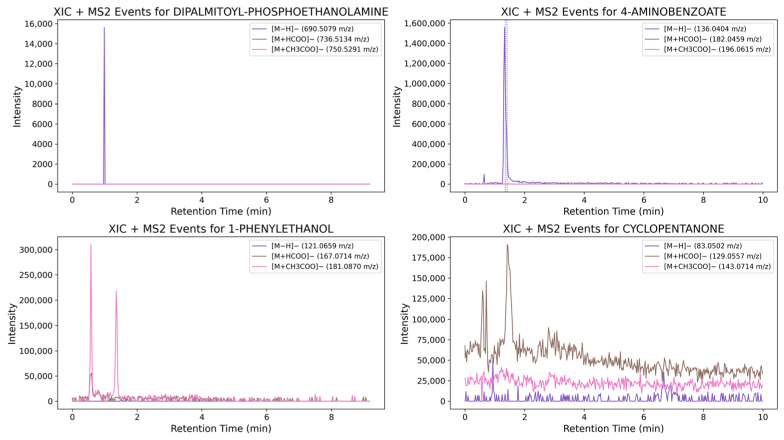
Examples of adduct searches for compounds not detected as [M+H]^+^ or [M−H]^−^. Representative outcomes are shown: no detectable signal (**top left**); detectable signal with MS/MS acquisition (indicated by the vertical line) but insufficient fragmentation quality for inclusion (**top right**); detectable signal without corresponding MS/MS data (**bottom left**); and signals indistinguishable from background noise (**bottom right**).

A total of 471 metabolites were included in the spectral library, leaving 132 of the 603 compounds in the MSMLS kit not represented. A summary of missing compounds is provided in Table 2, with representative examples shown in Figure A2 (Appendix A).

Cosine similarity scoring of library spectra against online resources at a threshold of 0.85 resulted in 924 spectra meeting the inclusion criteria, while 220 spectra fell below this threshold. Of these, 15 entries were removed due to insufficient spectral quality, including the presence of artifacts or low-intensity fragmentation signals. Additionally, 24 entries were replaced with spectra obtained from an orthogonal chromatographic method while retaining the original RT. Specifically, fragmentation spectra acquired using RPLC were incorporated into the HILIC library, and vice versa, when interference was observed but suitable spectra were available under alternative conditions using the same adduct. The remaining 165 entries were retained following manual inspection, either unchanged or annotated as containing minor interference.

The 132 compounds not included in the library were further evaluated by screening raw data for common adducts (+H, +Na, +K, +NH_4_^+^ in positive mode; −H, +HCOO^−^, +CH_3_COO^−^ in negative mode). Missing entries were categorized based on the absence of detectable signal, low-quality fragmentation spectra, interference from isobaric contaminants, or failure to trigger MS/MS acquisition despite detection at the MS1 level. The distribution of these categories across analytical methods is summarized in Table 2.

Across all analytical methods, the majority of missing compounds were attributed to the absence of detectable signal. Smaller proportions were associated with low-quality spectra, interference from contaminant signals, or failure to trigger MS/MS acquisition despite detection at the MS1 level.

**Figure 3:**
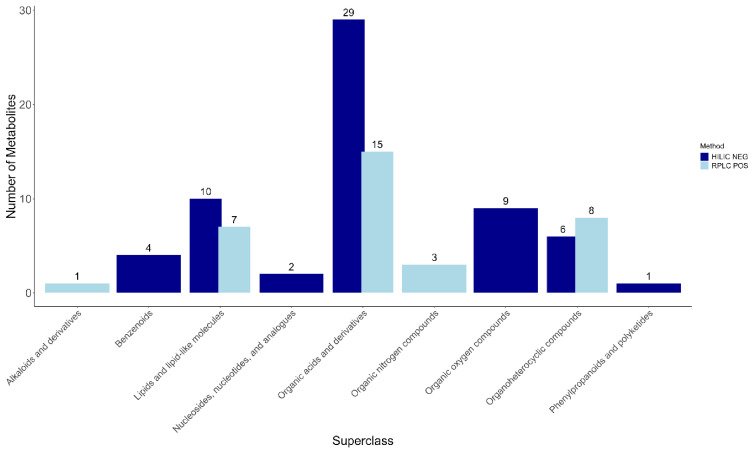
Distribution of identified metabolites by chemical superclass in the proof-of-concept serum analysis. Metabolites were classified according to the ClassyFire ontology [24]. Dark blue bars represent compounds detected using HILIC ESI−, while light blue bars represent compounds detected using RPLC ESI+.

A simple proof-of-concept evaluation was performed in which human serum samples were analyzed using two complementary methods, HILIC ESI− and RPLC-ESI^+^. This evaluation focused on annotation within a single full-scan injection, supported by five MS/MS injections generated using an inclusion list. It serves as a feasibility demonstration rather than a comprehensive assessment of acquisition robustness, which has been addressed previously in an independent validation paper. Spectral matching against the curated in-house library yielded 61 level 1 identifications using HILIC ESI− and 34 using RPLC ESI+. The distribution of identified metabolite superclass is shown in Figure 3.

**Figure 4:**
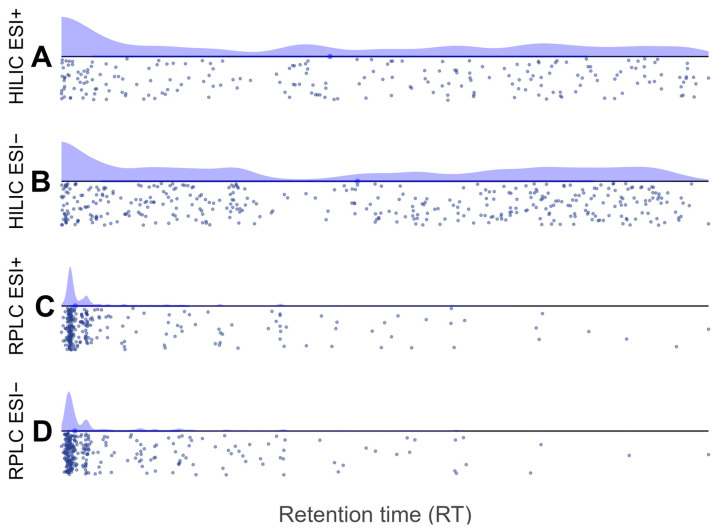
Distribution of RT for library compounds across chromatographic methods. Density plots and scatterplots illustrate the spread of compounds along the chromatographic gradient for (**A**) HILIC ESI+, (**B**) HILIC ESI−, (**C**) RPLC ESI+, and (**D**) RPLC ESI−.

The RT distributions of the detected metabolites across the four analytical methods are presented in Figure 4, illustrating differences in chromatographic behavior between HILIC and RPLC conditions.

## 4. Discussion

In this study, we developed a practical workflow for the construction of a mass spectral library using a structured mixture design based on molecular mass and logD. Using this approach, 471 of 603 metabolites (~78%) from the MSMLS reference standard kit were successfully detected and incorporated into the library, generating 1144 curated MS/MS spectra. The combination of orthogonal chromatographic and ionization methods provided substantial coverage, with 97.6% of detected metabolites captured by the complementary use of RPLC ESI+ and HILIC ESI−. Together, these results demonstrate that high-quality spectral libraries can be generated with reduced analytical burden while maintaining broad metabolite coverage.

Previous efforts to construct LC-Orbitrap-based spectral libraries have demonstrated the feasibility of generating high-quality MS/MS reference data from authentic standards (e.g., Phapale et al. 2021) [16]. However, these approaches typically do not explicitly address the experimental design required to optimize throughput and minimize co-elution during data acquisition. In contrast, the workflow presented here introduces a structured mixture design based on molecular mass and logD, enabling systematic grouping of compounds to reduce the number of injections while preserving chromatographic separation and spectral quality.

**Figure 5:**
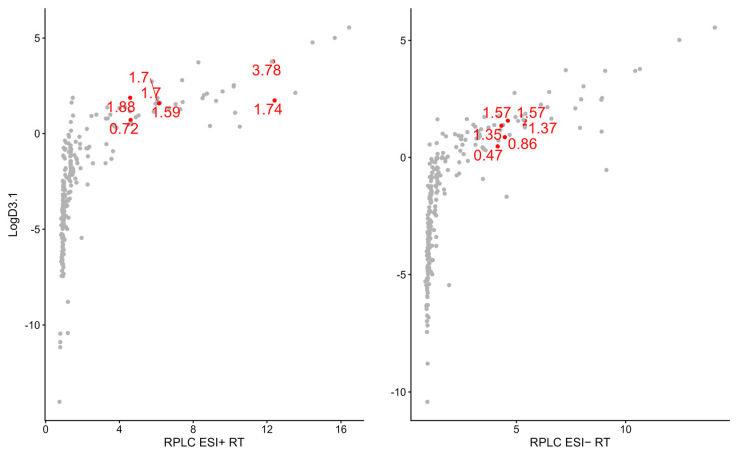
Scatterplot of calculated logD_3.1_ values against RT for compounds detected in reverse-phase LC-MS using ESI+ (**left**) and ESI− (**right**). A general trend of increasing retention with increasing logD is observed, although variability is present. Red point and label show co-elution of compounds placed in same injection mix. Red label shows calculated logD_3.1_.

The relationship between compound lipophilicity and chromatographic retention is well established, particularly in reverse-phase chromatography, where more lipophilic compounds exhibit increased retention. A minimum logD spacing of 0.15 was found to support mixtures of 15 compounds while maintaining sufficient chromatographic separation. In addition, comparison of theoretical logD_3.1_ values with experimental RT in the reverse-phase system reveals a general trend of increasing retention with increasing logD, providing an additional layer of quality control, as deviations from this trend may indicate co-elution, interference, or potential misannotation (Figure 5).

Figure 5 highlights the observed co-elution within individual mixtures in the reverse-phase methods. In total, 6 out of 147 compounds in positive ionization and 6 out of 207 compounds in negative showed co-elution with compound from the same mixture when using minimum 1 min retention and 0.2 min. tolerance as filters. In positive ionization, two of these pairs exhibited substantial differences in calculated logD_3.1_ values (0.72 vs. 1.88 and 1.74 vs. 3.78), indicating that co-elution may still occur despite significant differences in lipophilicity. The third pair showed a logD difference of only 0.11, reflecting a limitation in mixture design where compounds were placed closer than the intended spacing threshold. In negative ionization mode, co-eluting pairs showed logD differences of 0.20, 0.71, and 0.88, further demonstrating that while logD-based grouping reduces the likelihood of co-elution, it does not fully eliminate it. These observations underscore both the utility and the limitations of using logD as a guiding parameter for mixture design.

These initial results highlight the complementarity of the two chromatographic-ionization approaches. The higher number of identifications in HILIC ESI− underscores the value of this mode for polar metabolite profiling in serum, while RPLC ESI+ extends coverage to a broader range of compounds with different physicochemical properties. Together, the methods enable a more comprehensive mapping of the serum metabolome [25]. The complementary methods can be observed in Figure 4. The bottom two plots of the figure (RPLC ESI+/ESI−) indicate a high presence of polar compounds in the kit, as evidenced by their RT being at or near the injection peak of the method. Low RT for polar compounds in the reverse-phase system is also exacerbated by the UHPLC starting condition of 10% B mobile phase, which is relatively high, but chosen to reduce the chance of compound precipitation at the time of injection. The top two plots of Figure 4 (HILIC ESI+/ESI−) along with the coverage described in Figure 2 show that HILIC is well suited for this type of analysis both in chromatographic distribution and in number of compounds covered. Especially, HILIC coupled with ESI− performed at a high pH (approximately 9) effectively covers a greater number of metabolites in a single run compared to other methods, as supported by previous literature [26].

**Figure A3:**
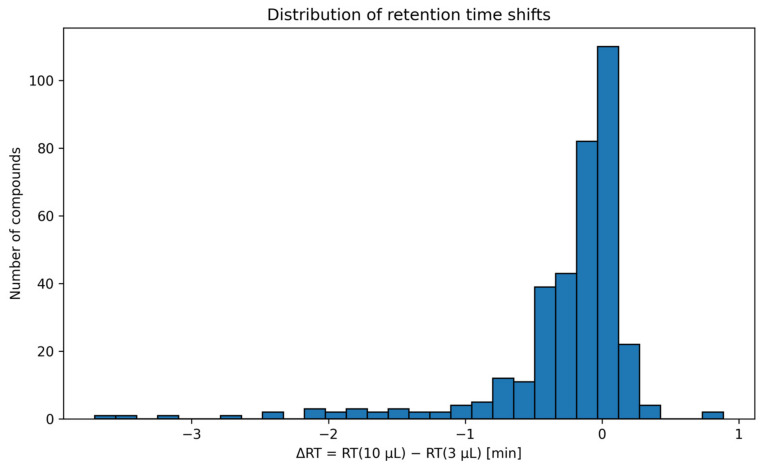
Distribution of ΔRT in HILIC ESI− between 3 µL and 10 µL injections. Negative ΔRT values indicate earlier elution in the 10 µL injection relative to the 3 µL injection, and vice versa. The largest RT differences are observed for negative ΔRT values, primarily associated with peak splitting. In these cases, the first peak exhibits a markedly reduced RT and a larger relative area under the curve (AUC), suggesting column overload.

Spectra included in the library from 10 µL injections that were not detected in the 3 µL injections (*n* = 52) are tagged using the library tagging function, as RT may differ at higher injection volumes. Matches to these entries should be considered level 2 identifications due to the uncertainty in RT, particularly for HILIC methods. Comparison of extracted ion chromatograms from both 3 µL and 10 µL injections across all compounds showed negligible RT differences for reverse-phase methods. In contrast, HILIC separations are more sensitive to injection volume, leading to noticeable RT shifts (Appendix A Figure A3). Therefore, additional caution is required when using RT as an identification parameter in HILIC systems [27].

To ensure high confidence in the spectral library, all reference standards were carefully curated. This process, although time-consuming, was facilitated by cosine similarity scoring using the matchms package, which enabled comparison of acquired spectra against public repositories such as GNPS and MoNA. A cosine similarity score of 0.85 was applied as a conservative empirical threshold to prioritize high-confidence matches for manual curation and inclusion in the library. However, cosine thresholds in MS/MS matching are dataset- and workflow-dependent; therefore, this value should be regarded as a practical quality-control criterion rather than a universal identification cutoff. Although not applied in this study, spectral entropy represents a complementary approach that incorporates the information content of spectra into similarity scoring and may further improve library quality assessment [28].

Comprehensive optimization of analytical conditions to maximize compound coverage was outside the scope of this work. It should be noted that library coverage is inherently method-specific, as only compounds that ionize under the applied conditions can be effectively detected and fragmented. Accordingly, inclusion of compounds that do not ionize in a given method is of limited practical value. Future efforts to extend library coverage could therefore focus on targeted re-injection of samples to obtain MS/MS spectra for compounds that were detected but not selected for fragmentation, for instance by increasing their concentration to enhance precursor ion intensity.

**Figure 6:**
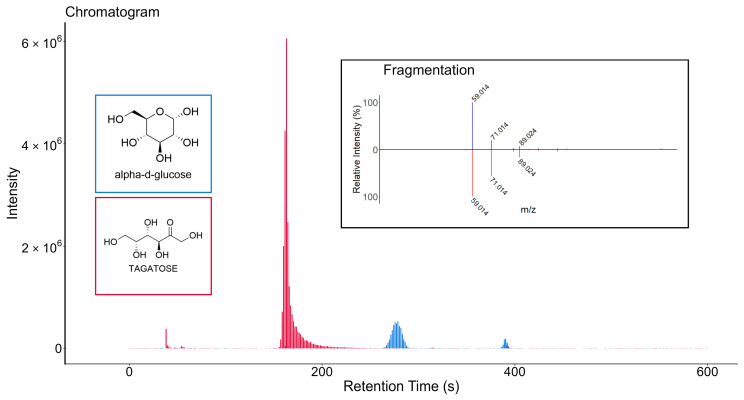
Extracted ion chromatogram and fragmentation of the two structural isomers alpha-d-glucose and tagatose (C_6_H_12_O_6_) captured with HILIC ESI−. Fragments and chromatography in blue show alpha-d-glucose while red show tagatose.

Reference standards add method-specific *m*/*z* and RT data to the experiment, enabling users to distinguish between compounds that might otherwise be difficult to discern based on fragmentation alone. Figure 6 illustrates alpha-D-glucose and tagatose, two of six very similar isomers in the MSMLS kit with regard to their fragmentation patterns, which require RT separation for unequivocal identification. These six metabolites have a predicted logD range from −2.76 to −2.93 at pH 3.1, resulting in significant chromatographic overlap in the reverse-phase library, with all having an RT of approximately one minute. However, in HILIC at pH 9, they are sufficiently separated for accurate identification.

This workflow should not be considered a plug-and-play solution. Method performance will depend on factors such as instrument platform, chromatographic setup, sample matrix, and analytical objectives, and therefore requires case-specific optimization when implementing in-house spectral libraries. Rather, this work provides a practical framework to improve throughput while maintaining data quality by incorporating distribution coefficient and RT information into mixture design, as well as fragmentation pattern matching against online mass spectral libraries to enhance curation confidence.

### Limitation

A true untargeted metabolomics analysis should be unbiased, meaning that the broad chemical diversity of such samples should be maintained in a library of standards. There were no observed differences between coverage of the superclasses contained in the MSMLS kit, but certain metabolites may not ionize well or may not be retained adequately in any of the methods used. Of the 603 metabolites in the kit, 132 are not represented here. An idea for increasing coverage in the future is to use the data of missing compounds and other low ionization efficiency compounds throughout these runs to make some higher concentration mixes rather than increasing the injection volume.

Metabolites producing sufficient fragmentation spectra for identification have been included in this library regardless of their respective RT. Some metabolites might co-elute, making it difficult to distinguish them based solely on RT and MS/MS spectra. This is especially true for the reported number of metabolites in the reverse-phase methods where several of the entries are contained within- or close to the injection peak.

The need for manual inspection of spectra, especially for borderline cases, introduces subjectivity and potential for human error. Validation should therefore be a continuous process in such work.

## 5. Conclusions

Here we report on a practical workflow for constructing an LC-HRMS mass spectral library of authentic standards for identification confidence. The result is a highly curated library covering 471 of the 603 unique standards present in the kit. HILIC ESI− and RPLC ESI+ cover 460 of these compounds, or 97.6%. Proof-of-concept injections identified 34 library metabolites in human serum for RPLC ESI+ and 61 for HILIC ESI−.

## Figures and Tables

**Table 1:** LC parameters. AmAc: Ammonium acetate, AmF: Ammonium formate, ACN: Acetonitrile, FA: Formic acid. * Measured prior to analysis.

Method	Mobile Phase A	Mobile Phase B	Flow Rate (mL/min)	Run Time (min)
HILIC ESI−	10 mM AmAc pH = 9 *	90% ACN with 10 mM AmAc pH = 9 *	0.45	15.2
HILIC ESI+	5 mM AmF pH = 3.1 *	95% ACN with 5 mM AmF + 0.1% FA	0.4	17
RPLC ESI−	5 mM AmF pH = 3.1 *	ACN + 0.1% FA	0.35	16.6
RPLC ESI+	5 mM AmF pH = 3.1 *	ACN + 0.1% FA	0.35	19.6

**Table 2:** Summary of reasons for exclusion of compounds not represented in the spectral library across chromatographic and ionization methods. Categories include absence of detectable signal, low-quality fragmentation spectra, interference from contaminant signals across chromatography, and detection at the MS1 level without corresponding MS/MS acquisition.

	HILIC ESI+	HILIC ESI−	RPLC ESI+	RPLC ESI−
No Signal	107	103	108	118
Low quality spectra	9	12	7	4
Contaminant signal	10	7	10	8
MS1 peak without MS/MS	6	10	7	2

## Data Availability

Exports of library data and proof-of-concept identifications are included in the Supplementary Materials. mzML files of library injections is available at https://doi.org/10.18710/NKTXLE (accessed on 5 June 2026).

## Supplementary Materials

The following supporting information can be downloaded at: https://www.mdpi.com/article/10.3390/metabo16060412/s1, SI_1_1_Compoundlist.csv—Entire compound list with mixture assignment of compounds. SI_1_2_RPLC__NEG.csv—Compounds detected in method RPLC_NEG. SI_1_3_HILIC_POS.csv—Compounds detected in method HILIC_POS. SI_1_4_HILIC_NEG.csv—Compounds detected in method HILIC_NEG. SI_1_5_RPLC_POS.csv—Compounds detected in method RPLC_POS. SI_1_6_RPLC.csv—proof-of-concept detected compounds in human serum for RPLC_POS. SI_1_7_HILIC.csv—proof-of-concept detected compounds in human serum for HILIC_NEG.

## Appendix A

**Table A1:** Compound Discoverer settings used for the feasibility demonstration. All parameters were set to template defaults, except for the RT tolerance used for database matching. Grouping and alignment settings were not applied, as the analysis was performed on five identification-only (MS/MS) files and a single sample file.

Step	Parameter	Value/Setting
Software	Version	Compound Discoverer 3.3
Workflow	Template	Untargeted Metabolomics with Statistics Detect Unknowns with ID Using Local Databases
Peak detection	Mass tolerance	5 ppm
	Minimum peak intensity	10,000 (instrument units)
	S/N threshold	1.5
	Remove baseline	Yes
MS/MS Matching	Library	mzVault
	Mass tolerance (precursor)	10 ppm
	Match ion activation energy	Match with tolerance (20)
	Use retention time	True
	RT tolerance	0.2 min (RPLC); 0.4 min (HILIC).
